# Lipedema: pathophysiological insights and therapeutic strategies – An update for dermatologists

**DOI:** 10.1016/j.abd.2025.501270

**Published:** 2026-01-08

**Authors:** Taciana Dal'Forno-Dini, Martina Souilljee Birck, Rafaela Malmann Saalfeld, Clayton Luiz Dornelles Macedo, Edileia Bagatin

**Affiliations:** a Serviço de Dermatologia, Hospital São Lucas/PUCRS, Porto Alegre, RS, Brazil; b Serviço de Dermatologia, Santa Casa de Misericórdia de Porto Alegre, Porto Alegre, RS, Brazil; c Graduação em Medicina, Pontifícia Universidade Católica do Rio Grande do Sul, Porto Alegre, RS, Brazil; d Departamento de Medicina do Esporte, Universidade Federal de São Paulo, São Paulo, SP, Brazil; e Departamento de Dermatologia, Universidade Federal de São Paulo, São Paulo, SP, Brazil

**Keywords:** Dermatology, Esthetics, Lipedema, Patient care team / Multidisciplinary care team

## Abstract

Lipedema is a chronic and progressive disorder characterized by disproportionate fat accumulation, mainly affecting the lower extremities of women, and commonly accompanied by sensations of heaviness, tenderness, and discomfort. While its pathogenesis remains largely unknown, genetic, hormonal, and microvascular factors have been implicated. The condition often coexists with psychological distress, which significantly detracts from the quality of life of affected individuals. Diagnosis is primarily clinical, as no specific biomarkers or imaging modalities have been proven sufficiently reliable for identification. Proposed managements are controversial, although current treatment focuses on symptom management and disease control through conservative methods such as compression and non-invasive device therapies, specialized diets, and physical rehabilitation or surgical treatments. Psychological support is vital in addressing the emotional challenges of the condition. Despite recent advancements in the understanding and management of lipedema, there remains a critical need for further research to establish standardized diagnostic criteria and targeted therapeutic strategies for this debilitating condition.

## Introduction

Lipedema is a chronic, progressive disorder of adipose tissue characterized by a symmetrical and disproportional subcutaneous fat accumulation, typically affecting one or more specific body regions, including the arms, lower abdomen, hips, buttocks, thighs, and lower legs. The condition was first described in 1940 as a clinical syndrome affecting women, marked by subcutaneous deposition of fat in the buttocks and lower extremities, and edema unresponsive to typical weight loss interventions.^1^ Despite its early identification, lipedema remained largely underrecognized for decades and was frequently misdiagnosed as obesity, lymphedema, venous insufficiency, or even cellulite. This lack of awareness contributed to delayed diagnoses and inappropriate treatments for many patients.^2^ Only recently, through the development of comprehensive clinical guidelines, such as the *German S2k Guidelines*, lipedema has been increasingly acknowledged as a distinct medical condition.^3^ In the *S2k Guideline*, lipedema is defined as a “painful, disproportionate symmetric distribution of adipose tissue of extremities occurring almost exclusively in women”.^3^ The most affected areas include the lower abdomen, hips, buttocks, thighs, and lower legs.^4^ Individuals with lipedema frequently report increased sensitivity to touch, pain, easy bruising, and a sensation of heaviness or fatigue in the affected limbs.^5^ Its onset is often associated with periods of hormonal fluctuation, such as puberty, pregnancy, or menopause.^6^, ^7^, ^8^

The reported prevalence of lipedema varies significantly among European countries, ranging from 0.06% to 39%. In Brazil, it is estimated that approximately 8.8 million women (about 12.3% of the female population) present symptoms highly suggestive of lipedema, frequently associated with comorbidities like hypertension, anemia, anxiety, and depression.^9^ Notably, body dissatisfaction and psychological distress have contributed to increased awareness and demand for diagnosis of this condition.^3^, ^10^

Nevertheless, lipedema continues to be underdiagnosed or misdiagnosed, often confused with conditions such as lymphedema, lipohypertrophy, cellulite (also known as gynoid lipodystrophy), or obesity.^10^, ^11^ Currently, there is no reliable biomarker or widely accessible diagnostic tool for lipedema; diagnosis is based primarily on clinical history and physical examination. Treatment focuses on alleviating pain and managing symptoms, aiming to improve quality of life.^12^

This study aims to support the development of an evidence-based treatment protocol while emphasizing the urgent need for further research to enhance understanding and management of this complex condition.

## Etiology and pathogenesis

The etiology of lipedema remains multifactorial and complex, reflecting the interplay of genetic, hormonal, and microvascular factors that are not yet fully elucidated. It has been reported that up to 60% of patients with lipedema have an affected first-degree relative with the same condition.^8^ Based on the analyses of familial clusters, in which the most affected family members are grandmothers and mothers, an autosomal dominant inheritance pattern with incomplete penetrance (sex limitation) may be suggested. Mutations in genes like *AKR1C1* (linked to adipogenesis and progesterone levels) and *PIT1* (involved in growth and sex hormones) have been found in affected families.^13^, ^14^ *AKR1C1* mutation is believed to reduce aldo-keto reductase activity, increasing levels of allopregnanolone (a potent analgesic) while also decreasing prostaglandin F2-alpha levels and raising progesterone levels, which stimulate adipogenesis.^15^

The disproportionate accumulation of adipose tissue in the lower body, particularly coinciding with periods of hormonal fluctuation, suggests that dysregulation of estrogen signaling plays a key role in the pathophysiology of lipedema. This appears to be associated with an imbalance in estrogen receptor expression within subcutaneous adipose tissue in affected regions. Estrogen exerts its effects primarily through two types of intracellular receptors: Estrogen Receptor alpha (ERα) and Estrogen Receptor beta (ERβ). These receptors regulate distinct sets of genes, often with opposing metabolic effects. ERα is generally associated with promoting adipogenesis and reducing lipolysis, while ERβ tends to have anti-adipogenic and protective effects against excessive fat accumulation. In individuals with lipedema, studies suggest an increased ERα/ERβ ratio in the subcutaneous adipose tissue of the lower body. This shift leads to reduced inhibitory influence from ERβ and enhanced activation of ERα-dependent pathways. As a result, there is upregulation of genes involved in lipid and glucose uptake, inhibition of lipolysis, and mitochondrial dysfunction, which collectively contribute to fat accumulation and metabolic alterations in the affected areas.^15^, ^16^

Furthermore, gene expression studies show increased aromatase *CYP19A1* ‒ the enzyme that converts androgens to estrogen ‒ in lipedema subcutaneous fat compared to healthy controls and even to the abdominal fat of the same patient. Additionally, estrogen has been found to induce *ZNF423*, a transcription factor also upregulated in lipedema. It is involved in preadipocyte differentiation via PPARG activation, suggesting a potential mechanism by which estrogen contributes to adipocyte hyperproliferation and fat accumulation in lipedema.^7^ Also, adipocytes in lipedema may exhibit enhanced local production of steroidogenic enzymes, further increasing local estrogen activity through ERα activation and perpetuating adipose tissue expansion.^15^

Recent multi-omics studies have identified metabolic, lipid, and gene expression abnormalities in lipedema. Key findings include disrupted lipid metabolism and increased sphingolipids, which may drive cell proliferation. Additionally, Bub1 was identified as a key regulator of abnormal Adipose-Derived Stem Cell (ADSC) growth, and its inhibition reduced proliferation, highlighting it as a potential therapeutic target and a reason for optimism.^17^

Recent insights into lipedema pathophysiology reveal immune and vascular dysfunctions within affected adipose tissue. Histology shows increased infiltration of CD45+, CD68+, and CD163+ immune cells, with a predominance of M2 macrophages, indicating chronic low-grade inflammation and tissue remodeling. Concurrently, early microangiopathy and increased endothelial permeability have been identified, even in initial disease stages, marked by disrupted tight junctions and altered endothelial behavior. These vascular changes may lead to interstitial fluid buildup and tissue hypoxia, further driving fibrosis and inflammatory signaling.^18^ Microvascular dysfunction – including increased capillary fragility and permeability – contributes to fluid leakage into the interstitial space, resulting in localized edema and a sustained inflammatory state. Inflammation is thought to drive the microangiopathy in lipedema and is perpetuated by elevated M2 macrophages, platelet factor 4, and lymphocytes. These microvascular changes are believed to play a crucial role in both the onset and progression of lipedema.^15^

**Figure 1:**
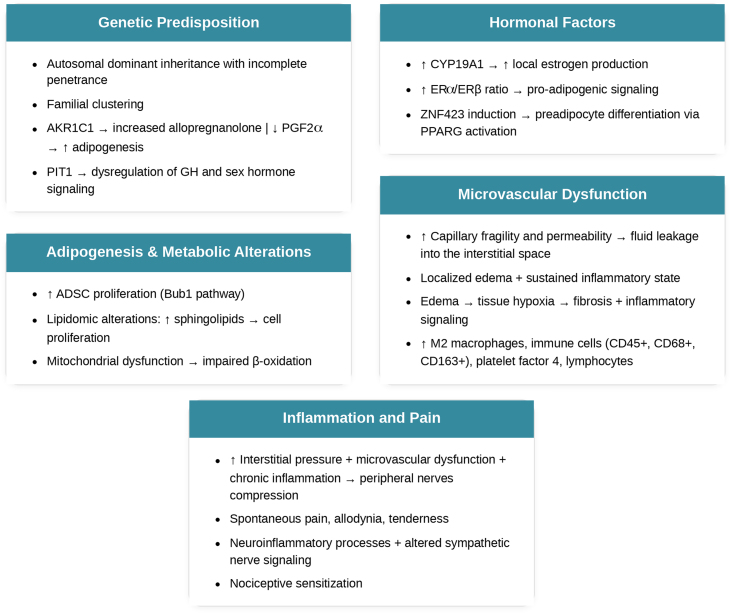
Pathogenic cascade of lipedema.

Additionally, pain in lipedema appears to be multifactorial. It is thought to arise from a combination of microvascular dysfunction, chronic inflammation, and increased interstitial pressure that may compress peripheral nerves. Patients often report spontaneous pain, allodynia, and tenderness, which are not fully explained by mechanical load or obesity alone. Neuroinflammatory processes and altered sympathetic nerve signaling have also been implicated in pain sensitization pathways. Collectively, these findings suggest that immune dysregulation, vascular fragility, and nociceptive sensitization interact to drive many of the hallmark symptoms of lipedema.^19^ Fig. 1 summarizes the pathogenic cascade of lipedema.

## Clinical manifestations

### Disproportionate fat distribution

Recent research consistently demonstrates that lipedema is characterized by a distinct and recognizable pattern of fat deposition. Patients typically present with bilateral and symmetrical enlargement of the legs, with a clear demarcation at the ankles ‒ commonly referred to as the “cuffing sign” ‒ while the feet and hands remain unaffected. This adipose tissue accumulation is notably resistant to conventional weight loss strategies and is frequently associated with joint hypermobility.^7^, ^12^

Symptoms often worsen throughout the day, particularly with heat exposure or prolonged standing, and are commonly accompanied by sensations of heaviness, tenderness, and discomfort in the affected areas.^10^, ^20^ Notably, the *S2k Guidelines* emphasize that “a disproportionate increase of adipose tissue on the extremities without corresponding symptoms shall not be diagnosed as lipedema”. Additionally, although morphological staging has historically been used, there is currently insufficient evidence to determine disease severity based solely on appearance, and no validated symptom-based staging system is available.^3^

**Figure 2:**
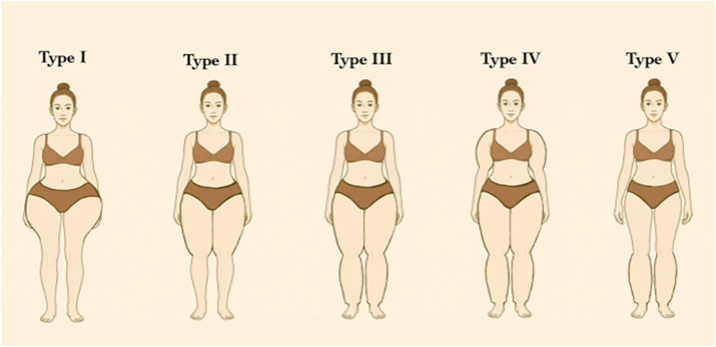
Graphical diagram representing the five subtypes of lipedema according to the distribution pattern.

**Figure 3:**
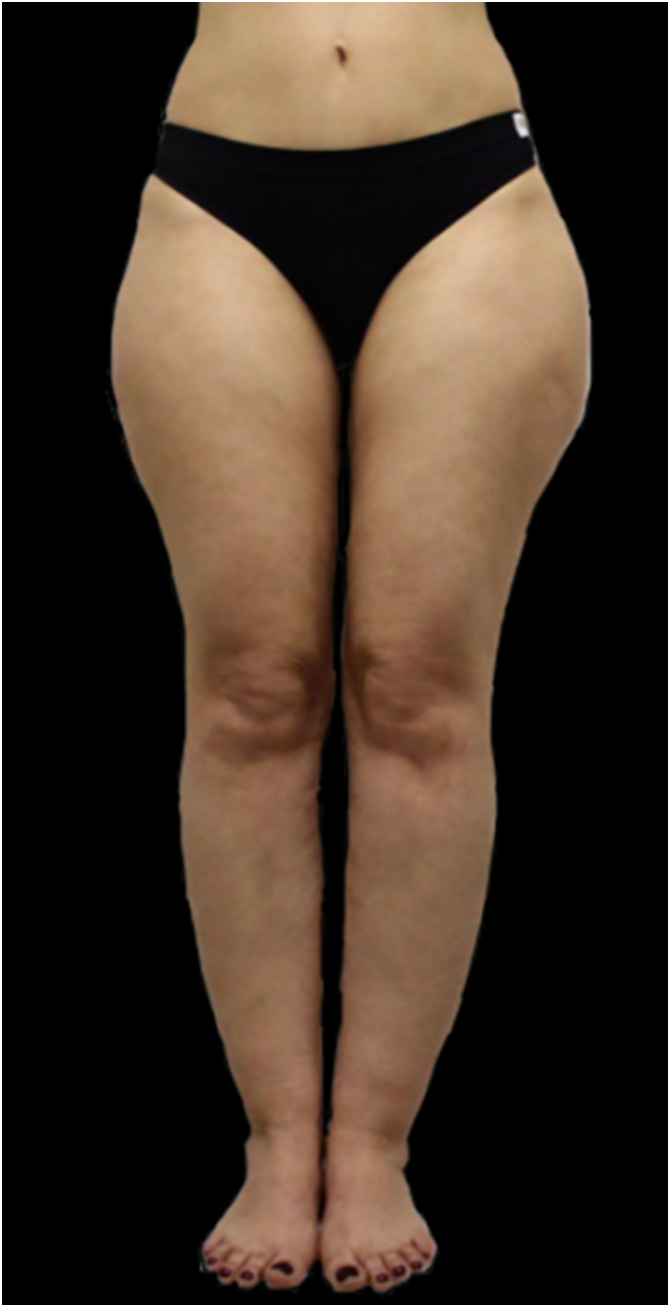
Lipedema, type I. Note the asymmetry between the limbs, with greater fat accumulation in the left lower limb.

**Figure 4:**
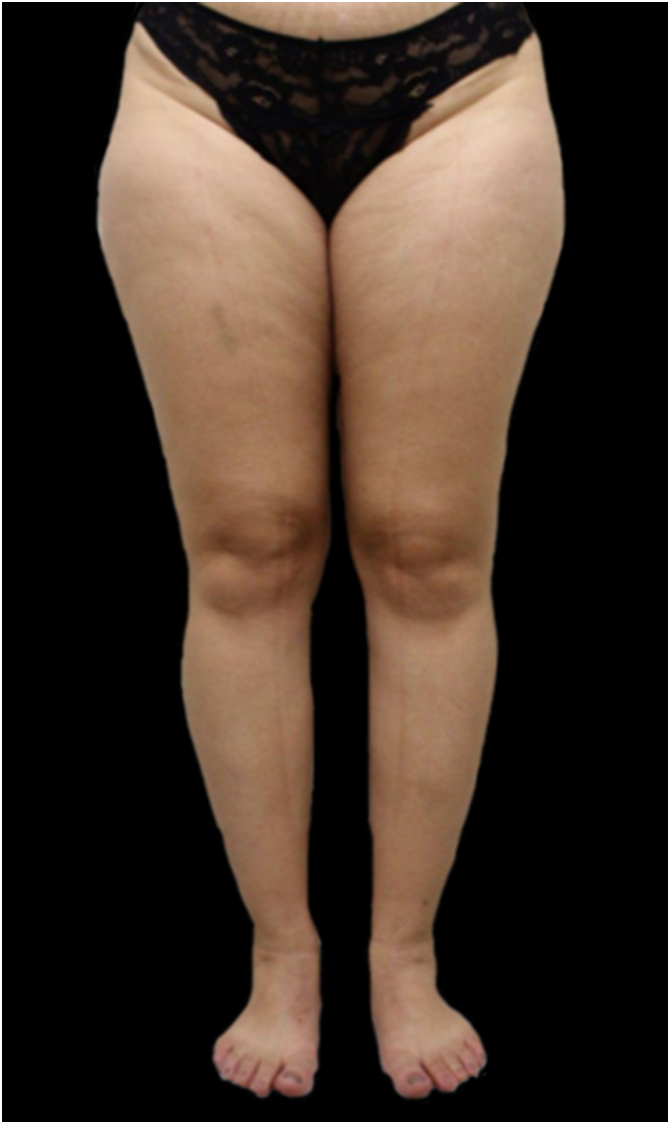
Lipedema, type II. Note the presence of spontaneous hematoma in the right lower limb.

**Figure 5:**
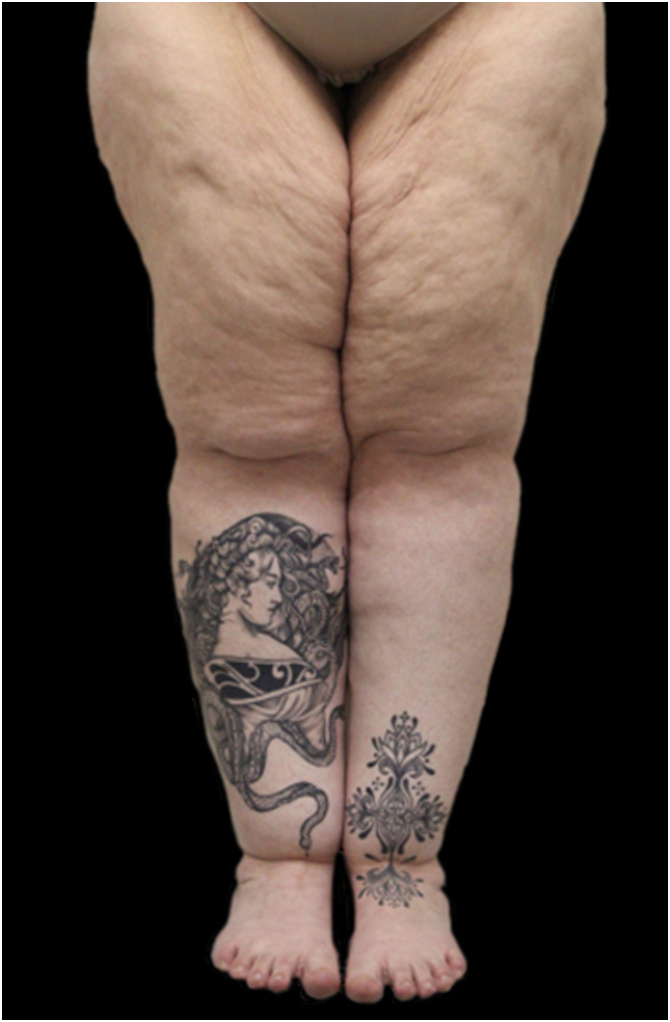
Lipedema, type III. The patient had tattoos made over the affected areas in an attempt to conceal the condition.

**Figure 6:**
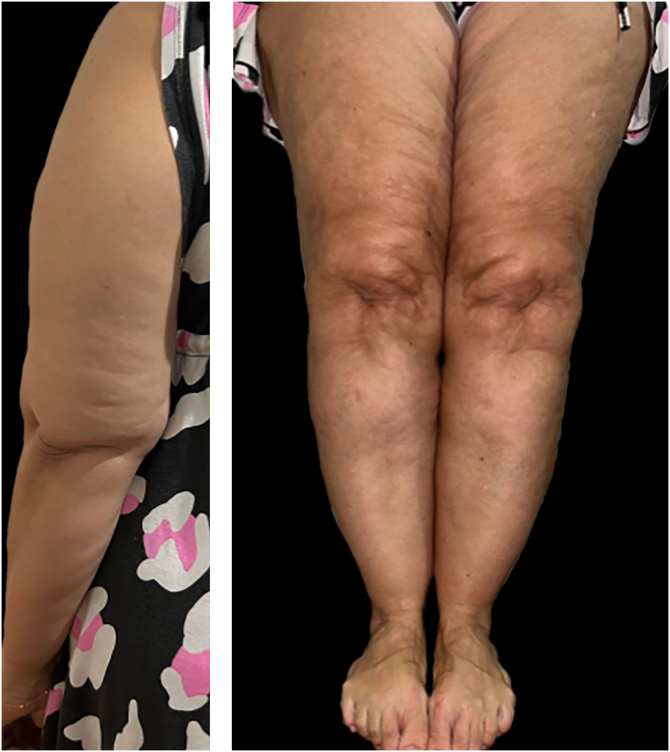
Lipedema, type IV.

Despite this, lipedema is commonly described based on two classification systems: distribution types and morphological stages. According to distribution patterns (Table 1), lipedema is categorized into five types (Fig. 2): Type I involves fat accumulation primarily around the buttocks, hips, and pelvic area (Fig. 3); Type II extends from the hips to the knees, often including the inner thighs (Fig. 4); Type III affects the entire lower limb from hips to ankles (Fig. 5); Type IV includes additional involvement of the arms (Fig. 6); and Type V, although less common, is characterized by fat distribution predominantly in the calves Notably, some literature also describes involvement of the lower abdomen, especially in Types I and II, although this is not consistently reflected across all classification systems.^8^, ^15^, ^21^, ^22^

**Table 1:** Classification of lipedema types based on anatomical fat distribution. Adapted from Amato et al (2021).

Type	Description
Type I	Fat accumulation primarily around the buttocks, hips and pelvic area
Type II	Fat distribution from hips to knees, often including the inner thighs and possibly the lower abdomen
Type III	Fat extends from hips to ankles, involving the entire lower limbs
Type IV	Involvement of the arms in addition to the lower body
Type V	Fat predominates in the calf region only (rare presentation)

Regarding morphological appearance, lipedema progresses through four clinical stages (Table 2): Stage I is characterized by smooth skin with thickened but soft subcutaneous tissue containing small nodules; Stage II presents with uneven skin surface due to the presence of larger nodules and increased fibrotic changes, often described as the “mattress phenomenon”; Stage III features significant fibrosis, hardening of the subcutaneous tissue, and the development of prominent, disfiguring fat lobules, particularly in the inner thighs and around the knees, with possible overhanging masses; and Stage IV corresponds to the development of secondary lipo-lymphedema, where lymphatic dysfunction becomes evident.^8^, ^15^, ^21^

**Table 2:** Lipedema clinical stages based on morphological changes of the subcutaneous tissue over time. Adapted from Duhon et al (2022).

Stage	Description
Stage I	Smooth skin surface with thickened but soft subcutaneous tissue containing small nodules; tissue has a spongy consistency
Stage II	Uneven skin surface due to larger nodules and the presence of fibrotic tissue; often associated with the “mattress phenomenon” (dimpled appearance)
Stage III	Significant fibrosis and hardening of the subcutaneous tissue; presence of large, disfiguring fat lobules, particularly in the thighs and knees, with overhanging masses
Stage IV	Development of the secondary lipo-lymphedema, characterized by combined fat deposition and lymphatic dysfunction, leading to swelling and edema

### Pain, tenderness and sensitivity

Pain is one of the most disabling features of lipedema. Patients frequently experience spontaneous pain and heightened tenderness in affected areas, even with minimal pressure.^19^ This increased perception of pain – often described as a constant, throbbing sensation – is thought to be linked to local inflammatory processes and possible micro-neuropathic changes within the subcutaneous tissue, leading to dysregulation of local-regional sensory nerve fibers.^12^

### Edema and microvascular abnormalities

Non-pitting edema is another common manifestation, particularly as the disease progresses, which can be primarily due to an underlying chronic venous disease. Furthermore, capillary fragility and livedo reticularis are present in loose connective tissue, leading to easy bruising in the patients affected.^15^, ^23^

However, it is important to note that the European Consensus Guidelines do not include edema in the diagnostic criteria for lipedema. It was posited that neither clinical examination nor diagnostic imaging showed a significant accumulation of fluid in the tissues of patients with lipedema, and therefore, the term “lipedema” was labeled as “outdated” and suggested that it could be renamed as “lipalgia syndrome”.^24^

### Metabolic and cardiovascular changes

Although lipedema is frequently associated with a high Body Mass Index (BMI), accumulating evidence suggests that its metabolic profile may differ from that typically seen in generalized obesity. In particular, women with lipedema appear to exhibit a relatively low prevalence of metabolic comorbidities such as diabetes and dyslipidemia, even in the presence of obesity-grade BMI values. For instance, studies have reported diabetes rates ranging from 2% to 6% among individuals with lipedema, whereas the prevalence in women with obesity in the general population is approximately 10%. This observation may be partially attributed to the predominance of gynoid fat distribution – localized to the hips, buttocks, and legs – which has been associated with reduced insulin resistance when compared to central or android fat accumulation.^15^, ^25^

Similarly, blood pressure measurements in women with early-stage lipedema tend to remain within normal ranges, with hypertension primarily observed in more advanced stages. Lipid profiles also appear to be relatively preserved, with total cholesterol levels ≥240 mg/dL reported in only a minority of cases (11.7%), contrasting with higher rates observed among women living with obesity without lipedema (33.5%). Taken together, the authors suggest that the adipose tissue in lipedema may confer a degree of cardiometabolic protection.^25^ However, more studies are needed to evaluate these findings.

Despite potential cardiometabolic advantages, the condition imposes significant clinical and functional burdens. Lipedema fat is resistant to conventional weight loss methods, often leading to further fat accumulation, mobility limitations, joint overload, and fatigue. Additionally, recent findings suggest that individuals with lipedema may present with altered vascular parameters, such as increased aortic stiffness, potentially associated with connective tissue changes secondary to hypermobility. While some cardiovascular risk markers may appear favorable in early stages, these findings underscore the need for further investigation into long-term cardiovascular outcomes and the full systemic impact of lipedema.^13^, ^15^, ^25^

## Psychological and quality-of-life impacts

The physical symptoms of lipedema, including chronic pain, functional limitations, and visible changes in body contour, have a profound impact on individuals suffering from this condition.^26^ Patients with lipedema frequently experience reduced quality of life, along with increased rates of anxiety, depression, and body image disturbances. The psychosocial impact is further compounded by the frequent misdiagnosis or underdiagnosis of the condition, which can delay appropriate care.^27^ Several studies have demonstrated that the quality of life in patients with lipedema is markedly reduced compared to age-matched controls.^15^, ^17^, ^28^ Some of the tools used included assessments of symptom severity, quality of life, satisfaction with life, psychological flexibility, and social connectedness. Multiple hierarchical regression analyses showed that a higher quality of life was associated with greater psychological flexibility and social connectedness. Thus, Functional Analytic Psychotherapy (Acceptance and Commitment Therapy), targeting psychological flexibility and social connectedness as key mechanisms of change, may be useful in treating women with lipedema.^28^ Furthermore, on a standardized measure of health-related quality of life, anxiety or depression was found in 42% of individuals with lipedema, and a history of eating disorders ‒ particularly anorexia and binge eating ‒ is also common, being identified in 74% of 100 participants in another study.^15^ The impact of psychological distress in this condition can be vastly underestimated, and early diagnosis and treatment may help reduce the impact of lipedema on mental health.^24^

## Imaging findings

Imaging modalities have become essential adjuncts in the evaluation of suspected lipedema, particularly when the clinical picture overlaps with other conditions. Among these, high-frequency ultrasound stands out as a widely accessible and informative tool, capable of detecting characteristic changes in the subcutaneous tissue, including increased hypodermal thickness, a reticular pattern of hyperechogenic fibrous septa, and echogenic nodules likely representing microfibrotic alterations. These features are typically bilateral and symmetrical, with preservation of dermal thickness and echogenicity. Notably, the absence of dermal edema or fluid collections helps distinguish lipedema from lymphedema. Recent studies have proposed clinically applicable and reproducible ultrasound cutoff values to support the diagnosis of lipedema. The authors identified the pretibial region as the most accurate anatomical site for measurement, followed by the thigh and lateral leg. Based on the findings by Amato et al. (2021), the suggested ultrasound thresholds for diagnosing lipedema were 11.7 mm for the pretibial region, 11.9 mm for the thigh, and 8.4 mm for the lateral leg, with corresponding sensitivities of 0.77–0.79 and specificities up to 0.96. These values were derived from ROC curve analysis in a single-center study of 89 women and have not yet been externally validated or standardized across centers. These measurements aim to enhance diagnostic precision and support the differentiation of lipedema from other conditions in clinical practice.^22^

Furthermore, Dual-Energy X-Ray Absorptiometry (DEXA), also known as bone densitometry, traditionally employed to assess bone density and body composition, has recently emerged as a potential auxiliary tool in the diagnostic process of lipedema. In a single-center case-control study, Buso et al. analyzed body composition in 222 women (74 with lipedema and 148 controls) using DEXA and proposed a diagnostic index based on the ratio of leg fat mass to total fat mass. An optimal cutoff value of 0.384 was identified as a potential indicator of lipedema, while values below 0.383 were considered useful for excluding this condition with reasonable confidence. In patients with a suggestive clinical presentation, but not fully compatible with established diagnostic criteria, DEXA may help distinguish lipedema from obesity by objectively quantifying regional fat distribution. When incorporated into a broader diagnostic algorithm, it may enhance diagnostic accuracy, particularly in borderline or ambiguous cases.^29^

## Differential diagnosis

**Figure 7:**
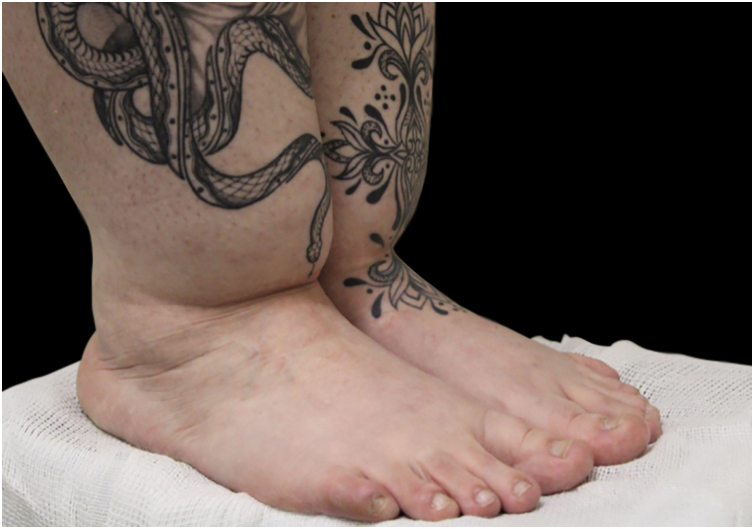
The cuffing sign.

Lipedema is frequently misdiagnosed due to its overlapping clinical presentation with several other disorders, including lymphedema, obesity, cellulite, lipohypertrophy, chronic venous insufficiency, and rare adipose tissue disorders such as Dercum’s disease and Madelung’s disease. A thorough differential diagnosis is therefore essential to ensure appropriate management (Table 3). One of the primary distinctions lies in the distribution and nature of the adipose tissue. Lipedema typically affects females and manifests as bilateral, symmetrical enlargement of the extremities ‒ particularly the legs ‒ while sparing the feet, resulting in a characteristic demarcation above the ankles. The so-called “cuffing sign” is a clinical hallmark of lipedema (Fig. 7). In contrast, lymphedema often begins in the distal extremities, involves the feet, and is commonly unilateral or asymmetrically bilateral. The presence of Stemmer’s sign (inability to pinch the skin on the dorsum of the toes) strongly suggests lymphedema but is absent in pure lipedema. Additionally, skin changes such as fibrosis, papillomatosis, and recurrent infections (e.g., erysipelas) further support a diagnosis of lymphedema.^8^, ^19^

**Table 3:** Differential diagnosis of lipedema. Modified from Kruppa et al. (2020) and Kumar et al. (2022).

Feature	Lipedema	Lymphedema	Obesity	Lipohypertrophy	Cellulite	Chronic venous insufficiency
Sex	Female	Both sexes	Both sexes	Mostly female	Almost exclusively female	Both sexes
Family history	Often positive	May be positive (primary), negative in secondary	Common	Possible	Common	Possible
Symmetry	Symmetric	Often asymmetric	Symmetric	Symmetric	Symmetric	Often asymmetric
Feet involvement	Spared	Involved	Possible	Spared	Spared	Involved
Edema	Variable (non-pitting)	Pitting, persistent	Pitting, improves with rest	Absent	If present, it is not related	Pitting, increases with standing, improves with leg elevation
Tenderness/ Pain	Present	Absent or mild	Absent	Absent	Generally painless	May have heaviness, aching, or cramps
Bruising	Frequent	Rare	Rare	Rare	Uncommon	Possible, but due to venous fragility or trauma
Fat distribution	Lower limbs, thighs, arms	Starts distally (feet), may be focal	Central / Generalized	Thighs and buttocks	Localized buttocks and thighs	No disproportionate fat deposition
Response to weight loss	No significant change	Not applicable	Responds to diet, exercise, surgery	No change	Partial improvement	Weight loss may lessen edema but not eliminate venous reflux
Skin changes	Skin typically smooth	Thickened, discolored, or warty	Normal	Normal	Dimpling, “orange peel” and “mattress” pattern	Hyperpigmentation, lipodermatosclerosis, venous ulcers in advanced stages

Distinguishing lipedema from obesity is equally important. Unlike obesity, which affects both sexes and typically presents with central fat accumulation, lipedema fat is more resistant to weight loss, diet, and bariatric surgery. Moreover, lipedema is often associated with pain, tenderness, and easy bruising, symptoms that are generally absent in obesity. Lipohypertrophy, a condition resembling lipedema in its fat distribution, differs in the lack of associated pain, edema, or hematoma tendency.^8^ Chronic venous insufficiency may present with limb swelling, varicosities, and hyperpigmentation, but these features typically improve with rest and elevation, unlike in lipedema.^30^

Advanced imaging techniques ‒ such as duplex ultrasound, lymphoscintigraphy, Magnetic Resonance Imaging (MRI), and Computed Tomography (CT) ‒ can assist in the diagnostic process by revealing distinct patterns of tissue involvement and venous or lymphatic function. Ultimately, an accurate diagnosis requires a comprehensive clinical evaluation in conjunction with imaging and exclusion of other possible conditions.^8^, ^19^

### Lipedema vs. cellulite

Although often underappreciated in the differential workup, cellulite, also known as gynecoid dystrophy, may also contribute to diagnostic confusion, particularly in early or mild cases of lipedema. Historically, lipedema has often been misclassified as a severe or atypical variant of cellulite, particularly within aesthetic and dermatologic literature prior to the early 2000s. For instance, a 2002 review discussing the classification of cellulite based on skin consistency referred to an “edematous type”, characterized by sensations of leg heaviness and soreness ‒ features that overlap with those of lipedema and likely contributed to diagnostic ambiguity.^31^ In some contexts, lipedema was even referred to as an “extreme form of cellulite”.^32^

This misclassification was likely influenced by shared epidemiological and clinical features, including female predominance, involvement of the lower body, and changes in subcutaneous adipose tissue. Nevertheless, it is now well established that lipedema and cellulite represent distinct clinical entities. Cellulite is defined by the presence of skin dimpling, producing the characteristic “orange peel” or “mattress-like” appearance. It primarily affects the thighs, buttocks, hips, and, occasionally, the abdominal region. Unlike lipedema, cellulite is typically painless.^33^, ^34^, ^35^ While lipedema is a progressive and painful condition involving subcutaneous fat accumulation, cellulite is considered a morphological alteration of the dermis and subcutaneous tissue, typically painless and largely aesthetic in nature. In an effort to improve clinical evaluation, Hexsel et al. developed and validated the Cellulite Severity Scale (CSS), a standardized tool that quantifies severity based on five morphological parameters: number and depth of skin depressions, surface appearance (such as the characteristic “orange peel” or “mattress” pattern), degree of flaccidity or sagging, and the traditional Nurnberger-Muller classification. Each criterion is scored from 0 to 3, yielding a total score between 1 and 15, which stratifies cellulite as mild, moderate, or severe. Accordingly, in this most adopted classification of cellulite severity in research settings, the presence of symptoms is not considered a criterion. This further reinforces the notion that cellulite and lipedema are clinically distinct entities.^36^

Histologically, cellulite-prone areas like the gluteal region show subcutaneous tissue organized into layers separated by fibrous septa, which are key to its pathogenesis. These collagen-rich septa connect the dermis to deeper fascia and vary in size and orientation. In women, they run perpendicularly to the skin, forming vertical partitions that create a honeycomb structure of fat lobules. When the inward pull of septa is outweighed by the outward pressure of enlarged fat lobules, the skin surface becomes uneven, leading to dimpling. In individuals with low BMI, this imbalance causes subtle depressions, but as BMI increases, fat lobules expand, and septal tension is further disrupted, worsening the dimpling. In some cases, fat may even herniate through weakened septa, although this is seen as a secondary effect rather than a primary cause of cellulite.^33^

Sexual dimorphism in the structure of fibrous septa helps explain why cellulite mainly affects women. In men, septa form a crisscross pattern at oblique angles, offering greater support and reducing fat protrusion. They are also typically stronger, more numerous, and associated with smaller, evenly distributed fat lobules ‒ factors that minimize skin irregularities even with higher adiposity. In contrast, women have fewer, vertically oriented septa and larger, elongated fat lobules, making their dermal-subcutaneous interface more susceptible to cellulite.^33^

## Treatment and clinical management

Lipedema is currently considered a chronic disorder with progressive characteristics rather than a classical disease entity. Therapeutic approaches aim primarily at symptom reduction and prevention of disease progression. To date, no specific etiological treatment has been described.^8^, ^13^ Therefore, the main pillars of lipedema management are conservative treatments and surgical or other non-conservative interventions.

### Conservative treatment approaches

Physical activity and rehabilitation: Regular physical activity reduces proinflammatory adipokines and macrophages and improves blood flow, thereby counteracting hypoxia in adipose tissues.^24^ Exercise routines should be tailored to individual needs and ideally supervised by qualified health professionals.^12^ Beneficial exercises – which should start slowly and progress as tolerated – include swimming, aquatic therapy, elliptical machines, yoga, stationary biking, whole-body vibration, and walking.^15^ The goals of physical activity are weight control, improved muscle strength and mobility, and enhanced self-esteem.^20^

Special diets: Since lipedema is considered a polygenic disorder associated with chronic low-grade inflammation, some authors recommend anti-inflammatory diets such as the Mediterranean or ketogenic diet to reduce symptoms.^37^ Dietary management should prioritize reducing hyperinsulinemia and insulin resistance. Professional guidance from a nutritionist or a nutrologist physician is essential to ensure adherence and prevent relapse.^20^

Compression therapy: Combined decongestive therapy (CDT) integrates manual lymphatic drainage, compression, exercise, and skincare. CDT comprises two phases: phase 1 includes education, skin care, manual lymphatic drainage, and multilayer non-elastic compression bandaging; phase 2 involves continuation of phase 1 plus self-massage and compression garments. CDT primarily aims to reduce pain but does not prevent fat accumulation or disease progression. Flat-knit compression stockings are preferred due to their higher bending stiffness, allowing better support of deep tissue folds without constriction, and are more comfortable.^3^, ^24^ Compression therapy should be tailored to disease stage and individual tolerance. In the initial or more edematous phases, non-elastic multilayer compression bandages are often indicated to achieve effective volume reduction and tissue stabilization before transitioning to maintenance garments. These short-stretch bandages provide high working pressure and low resting pressure, promoting lymphatic return and reducing orthostatic edema. Once limb volume has stabilized, flat-knit compression stockings are recommended for long-term management, as their firm, inelastic structure provides consistent containment of the subcutaneous tissue while minimizing constriction and rolling. The compression class should be selected according to the degree of edema and fibrosis: class II (23–32 mmHg) is generally appropriate for stage I–II lipedema, while class III (34–46 mmHg) may be required for stage III disease with tissue induration or concomitant lymphedema. Proper fit and donning technique are essential to ensure efficacy and comfort, and periodic reassessment is recommended to adapt compression strength as clinical features evolve.^20^

Pharmacological therapy: Although no pharmacological therapies have been officially approved specifically for lipedema, recent advances in the understanding of its pathophysiology and its overlap with metabolic mechanisms have prompted the off-label use of anti-obesity medications, aiming to reduce inflammation and the volume of functionally impaired adipose tissue. Glucagon-Like Peptide-1 (GLP-1) and GLP-1 / Glucose-dependent Insulinotropic Polypeptide (GIP) receptor agonists (semaglutide, liraglutide, tirzepatide) suppress appetite, improve glycemic control, induce significant weight loss, and may modulate tissue inflammation. Emerging evidence suggests that weight reduction achieved with GLP-1 receptor agonists may relieve pain, joint pressure, and edema associated with lipedema. Case reports and observational data indicate improvements in quality of life, edema, and pain ‒ particularly with tirzepatide, which has been associated with weight losses exceeding 20% in some patients.^38^ While traditional weight loss strategies tend to have limited effects on lipedematous fat, GLP-1 agonists have demonstrated benefits in a retrospective cohort, including pain relief, reduced limb volume, and improved physical function, even when lipedematous fat is not entirely resolved.^39^ The use of anti-obesity pharmacotherapy in lipedema should be considered, particularly in cases of lipolymphedema or coexisting obesity, refractory pain and functional limitation, failure to respond to intensive lifestyle modification, and as preoperative preparation for surgical intervention. Although randomized controlled trials are still needed, clinical experience points to a promising therapeutic horizon for women with lipedema, through a transdisciplinary approach that integrates pharmacological treatment, physiotherapy and exercise, nutritional therapy, psychological support, and aesthetic or surgical procedures.^37^, ^39^

Long-term use of diuretics is discouraged because they do not address the underlying inflammatory processes. Metformin may be considered for patients with metabolic complications due to its potential to inhibit hypoxia-induced fibrosis in adipose tissue.^40^ Oral analgesics may be used for pain management, although systematic data on pharmacological therapies are lacking.^8^

Despite its association with sex hormones, there is no scientific basis for the use of estrogens, progestins, testosterone, or hormonal implants in the treatment of lipedema. These therapies may worsen the condition by stimulating adipose tissue growth or causing uncontrolled changes in body composition. The use of hormonal implants containing compounds such as gestrinone or testosterone has been inappropriately employed in aesthetic practice without any evidence of benefit for lipedema and with potential cardiovascular, metabolic, and endocrine risks.^39^

### Non-invasive interventions

Although liposuction seems to be the surgical method of choice for the reduction of the affected subcutaneous tissue of lipedema, it is also accompanied by a significant risk of complications. Adverse effects may include postprocedural pain, infection, prolonged recovery, scarring, bruising, ecchymosis, or edema, along with substantial financial costs. Nowadays, non-invasive devices have been confirmed as safer, more affordable methods for the reduction of localized fat deposits. There are five leading non-invasive techniques currently being used to treat subcutaneous adipose tissue deposits: Low-level laser therapy (LLLT), cryolipolysis, radiofrequency (RF), high-intensity focused ultrasound (HIFU), and shock wave therapy (SWT).^41^

LLLT: This therapeutic approach uses LLLT in the 635–680 nm wavelength range to promote adipocyte membrane permeability, allowing for the release of intracellular lipids without cell destruction. In the study by Savoia et al., LLLT was applied to areas including the hips, thighs, abdomen, and knees in patients with localized adiposity and cellulite. The results demonstrated a significant reduction in circumference in treated areas ‒ up to 2.15 cm in the abdomen and 1.87 cm in the thighs ‒ after a cycle of treatment sessions. Additionally, improvements in skin firmness and texture were observed, with high levels of patient satisfaction and no reported adverse effects. These findings support LLLT as a safe and effective noninvasive technique for body contouring and cellulite treatment, but there are no studies with lipedema.^42^

Cryolipolysis: This device therapy is a non-invasive technique that uses controlled cooling to selectively target and induce apoptosis of subcutaneous adipocytes by crystallizing intracellular lipids. These crystallized lipids are subsequently recognized as “foreign bodies” by the immune system, triggering localized panniculitis and apoptosis of the affected cells without damaging surrounding tissues. Several case reports have suggested that cryolipolysis may be an effective non-invasive option for lipedema treatment, reporting symptom improvement in small patient cohorts.^43^ According to a systematic review by Ingargiola et al. (2015), cryolipolysis has demonstrated efficacy in noninvasively reducing subcutaneous fat, with studies reporting an average fat reduction of 14.67% to 28.5% across treated areas such as the abdomen and flanks. The procedure is generally well tolerated, with high patient satisfaction and a low incidence of mild, transient adverse side effects like erythema, edema, and sensory changes.^44^ Despite its success in treating localized adiposity, the application of cryolipolysis for lipedema remains under-investigated. The pathophysiology of lipedema differs from simple localized fat accumulation, involving inflammatory components and microvascular abnormalities, which might influence treatment outcomes. Therefore, although cryolipolysis represents a promising adjunct therapy for symptom management and fat reduction in lipedema patients, more specific clinical trials are needed to establish its efficacy and safety in this population.^45^

RF: This technology employs high-frequency electromagnetic waves to induce thermal effects in subcutaneous tissues, leading to adipocyte disruption and gradual fat reduction. The mechanism involves deep tissue heating, which may promote lipolysis through sympathetic stimulation and increased metabolic activity. Clinical studies have shown reductions in abdominal circumference and fat thickness following RF treatment, with minimal side effects such as mild erythema and transient discomfort. However, while initial results are promising, limitations include small sample sizes and short follow-up durations, emphasizing the need for more robust and long-term clinical trials to validate efficacy and safety.^46^, ^47^ There are no studies evaluating RF for lipedema treatment.

HIFU: This noninvasive lipolysis technique uses focused ultrasonic energy to target adipose tissue, inducing thermal coagulation and mechanical disruption of adipocytes while preserving surrounding structures. It has gained popularity for body contouring applications, particularly in the abdominal, waist, and flank regions.^48^, ^49^ Several commercially available devices utilize HIFU technology, i.e. micro- and macro-focused ultrasound to deliver thermal energy to specific dermal and subcutaneous layers. Clinical studies report its efficacy in skin tightening, collagen remodeling, and fat reduction, with significant improvement in skin elasticity and lifting effects.^50^, ^51^ Clinical studies report favorable outcomes in terms of fat reduction and skin tightening; however, discrepancies between subjective patient assessments and objective clinical findings have been noted. Adverse events are generally mild and transient, including erythema, mild burning sensations, and occasional blistering, all of which typically resolve spontaneously without lasting sequelae.^48^, ^49^ There are no studies evaluating HIFU for lipedema treatment.

SWT: This is a non-invasive localized treatment method that utilizes either radial or focused acoustic waves to promote dermal remodeling. The initial mechanical stimulus by the pressure peaks induces cellular changes through mechanotransduction, mobilizing the extracellular matrix. This process promotes neocollagenesis and collagen remodeling, enhances microcirculation, and supports lymphatic drainage.^35^ In addition to improving skin elasticity and the appearance of cellulite, acoustic waves may also help reduce localized adiposities through mechanical disruption and improved tissue structure.^34^ A meta-analysis conducted by Knobloch and Kraemer (2015) evaluated 11 clinical studies – including 5 randomized controlled trials involving a total of 297 female patients with cellulite – and demonstrated that both radial and focused extracorporeal SWT significantly reduced cellulite severity. Treatment protocols typically included 6 to 8 sessions, delivered once or twice weekly, with outcomes assessed via standardized photography, circumference measurements, and ultrasonographic analysis. The authors reported improvements in skin texture, reduction in tissue edema, and increased dermal elasticity. Although the methodological quality of the included studies was variable (scores ranging from 22 to 82), the overall findings support the clinical efficacy of extracorporeal SWT in aesthetic indications.^52^ While the data pertain specifically to cellulite, the underlying mechanisms – improved lymphatic flow, microvascular function, and connective tissue remodeling – are also pathophysiologically relevant to lipedema. In this context, a recent pilot study evaluated 15 patients with stage II lipedema with the combined use of defocused and radial shock wave therapy, mesotherapy, and *Kinesio Taping* (elastic therapeutic taping applied to the skin to reduce pain). The mesotherapy solution consisted of Lymdiaral® (Pascoe), a homeopathic compound containing Conium D3 (2.5 mg), Hydratis D3 (2.5 mg), Viscum album D2 (2.5 mg), Phytolacca D4 (2.0 mg), Scilla D1 (2.0 mg), and sodium chloride. This study reported reductions in limb circumference, pain, and tissue stiffness, along with improved quality of life. However, the study’s lack of a control group, small sample size, short follow-up, and the use of multiple simultaneous therapies limit the ability to attribute the observed benefits specifically to shock wave therapy.^53^ Thus, the SWT appears to be a promising therapeutic approach for lipedema, warranting further investigation through controlled trials.

## Surgical interventions

Lipedema reduction surgery is currently the sole technique for eliminating abnormal lipedema tissue, including adipocytes, nodules, fibrotic extracellular matrix, and other non-adipocyte components. Besides, it is the only treatment known to slow the progression of lipedema and is ideally performed before complications and functional impairments arise.^15^

### Tumescent liposuction

For patients who do not respond sufficiently to conservative treatments, tumescent liposuction has emerged as a reliable surgical option. The German S2k Guideline recommends that it should be performed with a tissue and lymph-vessel conserving method, in 1 to 4 sessions on both legs, 1 or 2 sessions on both arms, and a maximum aspiration volume of 10% of the body weight. Moreover, immediately after the surgical procedure, CDT should be performed.^3^

According to the *European Lipedema Forum,* liposuction is effective only when patients are appropriately selected. Key criteria include: 1) Persistent symptoms despite 12-months of comprehensive conservative treatment; 2) Significant functional limitations, such as reduced mobility; 3) Stable body weight for at least 12-months to reduce the risk of postoperative weight regain; 4) Preoperative psychological evaluation to rule out eating disorders or mental health conditions that may affect outcomes; and 5) A BMI of 35 kg/m^2^ or lower.^24^ Liposuction is not recommended for individuals with a BMI exceeding 35 kg/m^2^ who also present with central obesity (waist-to-height ratio [WHtR] > 0.5). However, in rare cases where central obesity is not present, liposuction may still be considered for individuals with a higher BMI.^3^, ^37^, ^40^ Although many studies reported improving the disease's symptoms, such as reduction of spontaneous pain, bruising, and mobility impairment, there is still not enough evidence to support liposuction as the gold standard in treating lipedema.^37^, ^54^ It is also important to highlight that liposuction carries risks and potential complications, including post-procedural pain, infection, prolonged recovery, scarring, bruising, ecchymosis, edema, and substantial financial costs, which must be carefully considered in clinical decision-making.^54^

### Bariatric surgery

Bariatric surgery may be considered for individuals with lipedema and a BMI ≥ 40 kg/m^2^, and potentially for those with a BMI between 35–40 kg/m^2^. Recent studies report significant benefits in patients with lipedema and severe obesity following surgery. In cases with BMI 35–40 kg/m^2^, assessing the WHtR can offer additional guidance. A WHtR below 0.5 suggests low metabolic risk, indicating that bariatric surgery may not be necessary for these patients.^24^

## Psychosocial and emotional support

Addressing the psychological aspects of lipedema involves a multifaceted approach. Cognitive behavioral therapy focuses on modifying negative thought patterns and behaviors, thereby reducing symptoms of anxiety and depression; it can help patients develop effective coping strategies to manage the emotional challenges posed by lipedema. On the other hand, acceptance and commitment Therapy encourages patients to accept their experiences without judgment and commit to actions aligned with their values, fostering improved mental health outcomes. This approach has shown promise in enhancing psychological flexibility and emotional regulation in women with lipedema.^26^, ^28^

A holistic approach to lipedema treatment acknowledges the interplay between physical and psychological health. Incorporating psychological therapies alongside medical and physical interventions can lead to more comprehensive and effective management of the condition. Healthcare providers are encouraged to assess lipedema patients' mental health needs routinely and facilitate appropriate referrals to mental health professionals when necessary.^17^

## Role of the dermatologist

Dermatologists play a central role in the diagnosis and management of lipedema, as the condition often presents initially with cutaneous and subcutaneous manifestations that mimic other dermatologic or vascular disorders. Their expertise in recognizing patterns of adipose tissue distribution, assessing skin changes, and distinguishing lipedema from other disorders is critical for early and accurate diagnosis. Moreover, dermatologists are essential in coordinating multidisciplinary care, which includes vascular specialists, endocrinologists, nutritionists, physiotherapists, and mental health professionals. In clinical practice, dermatologists guide conservative treatment strategies ‒ such as compression therapy, skincare, and non-invasive device-based approaches ‒ and are frequently involved in the pre- and postoperative care of patients undergoing liposuction or aesthetic interventions. By integrating diagnostic precision with therapeutic and psychosocial management, dermatologists contribute significantly to improving outcomes and quality of life in patients with lipedema.

## Conclusion

Lipedema is a complex and often misunderstood condition, recently recognized as a distinct clinical entity. It affects many women and causes a significant physical and psychological impact. Despite growing awareness and advances in understanding, diagnosis remains difficult due to the absence of specific biomarkers and standardized criteria. While some treatments offer symptom relief, they do not stop disease progression, making surgical options like tumescent liposuction viable for many. Effective management requires a multidisciplinary approach combining medical care, rehabilitation, and psychological support. Future research should focus on clarifying its mechanisms, improving diagnosis, and developing targeted, innovative therapies to enhance outcomes and quality of life.

## ORCID ID

Taciana Dal’Forno Dini: 0000-0003-0848-9042

Martina Souilljee Birck: 0000-0001-9281-7744

Rafaela Malmann Saalfeld: 0009-0008-0157-0300

Clayton Luiz Dornelles Macedo: 0009-0006-9058-9147

Edileia Bagatin: 0000-0001-7190-8241

## Research data availability

The entire dataset supporting the results of this study was published in this article.

## Financial support

None declared.

## Authors' contributions

Taciana Dal’Forno-Dini: Design and planning of the study; collection of data, or analysis and interpretation of data; drafting and editing of the manuscript or critical review of important intellectual content; collection, analysis and interpretation of data; effective participation in research orientation; critical review of the literature; approval of the final version of the manuscript.

Martina Souilljee Birck: Design and planning of the study; collection of data, or analysis and interpretation of data; drafting and editing of the manuscript or critical review of important intellectual content; collection, analysis and interpretation of data; critical review of the literature; approval of the final version of the manuscript.

Rafaela Malmann Saalfeld: Design and planning of the study; collection of data, or analysis and interpretation of data; drafting and editing of the manuscript or critical review of important intellectual content; collection, analysis and interpretation of data; critical review of the literature; approval of the final version of the manuscript.

Clayton Luiz Dornelles Macedo: Design and planning of the study; collection of data, or analysis and interpretation of data; drafting and editing of the manuscript or critical review of important intellectual content; collection, analysis and interpretation of data; effective participation in research orientation; critical review of the literature; approval of the final version of the manuscript.

Edileia Bagatin: Drafting and editing of the manuscript or critical review of important intellectual content; effective participation in research orientation; critical review of the literature; approval of the final version of the manuscript.

## Conflicts of interest

None declared.

## References

[1] Wold L.E., Hines E.A., Allen E.V. (1951). Lipedema of the legs: a syndrome characterized by fat legs and edema. Ann Intern Med..

[2] Lause M., Kamboj A., Fernandez Faith E. (2017). Dermatologic manifestations of endocrine disorders. Transl Pediatr..

[3] Faerber G., Cornely M., Daubert C., Erbacher G., Fink J., Hirsch T. (2024). S2k guideline lipedema. J Dtsch Dermatol Ges..

[4] Hardy D., Williams A. (2017). Best practice guidelines for the management of lipoedema. Br J Community Nurs..

[5] Paula A.C.P. de, Oliveira J. de (2024). Lipedema: clinical characteristics, complications, and the importance of evidence-based practice. Rev Assoc Med Bras..

[6] World Health Organization (2024). International statistical classification of diseases and related health problems (11th revision) [Internet].

[7] Poojari A., Dev K., Rabiee A. (2022). Lipedema: insights into morphology, pathophysiology, and challenges. Biomedicines..

[8] Kruppa P., Georgiou I., Biermann N., Prantl L., Klein-Weigel P., Ghods M. (2020). Lipedema-Pathogenesis, diagnosis, and treatment options. Dtsch Arztebl Int..

[9] Amato A.C.M., Amato F.C.M., Amato J.L.S., Benitti D.A. (2022). Prevalência e fatores de risco para lipedema no Brasil. J Vasc Bras..

[10] Oliveira J. de (2024). Lipedema: a new phenomenon for many people and a new field of study for psychiatry, nutrition, and psychology in Brazil. Rev Assoc Med Bras..

[11] Lomeli L.D., Makin V., Bartholomew J.R., Burguera B. (2024). Lymphedema vs lipedema: similar but different. Cleve Clin J Med..

[12] Dudek J.E., Białaszek W., Gabriel M. (2021). Quality of life, its factors, and sociodemographic characteristics of polish women with lipedema. BMC Womens Health..

[13] van la Parra R.F.D., Deconinck C., Pirson G., Servaes M., Fosseprez Ph. (2023). Lipedema: what we don’t know. J Plast Reconstr Aesthet Surg..

[14] Duhon B.H., Phan T.T., Taylor S.L., Crescenzi R.L., Rutkowski J.M. (2022). Current mechanistic understandings of lymphedema and lipedema: tales of fluid, fat, and fibrosis. Int J Mol Sci..

[15] Herbst K.L., Kahn L.A., Iker E., Ehrlich C., Wright T., McHutchison L. (2021). Standard of care for lipedema in the United States. Phlebology..

[16] Katzer K., Hill J.L., McIver K.B., Foster M.T. (2021). Lipedema and the potential role of estrogen in excessive adipose tissue accumulation. Int J Mol Sci..

[17] Ishaq M., Bandara N., Morgan S., Nowell C., Mehdi A.M., Lyu R. (2022). Key signaling networks are dysregulated in patients with the adipose tissue disorder, lipedema. Int J Obes..

[18] Strohmeier K., Hofmann M., Jacak J., Narzt M.S., Wahlmueller M., Mairhofer M. (2022). Multi-level analysis of adipose tissue reveals the relevance of perivascular subpopulations and an increased endothelial permeability in early-stage lipedema. Biomedicines..

[19] Aksoy H., Karadag A.S., Wollina U. (2021). Cause and management of lipedema‐associated pain. Dermatol Ther..

[20] Forner-Cordero I., Forner-Cordero A., Szolnoky G. (2021). Update in the management of lipedema. Int Angiol..

[21] Ernst A.M., Bauer H., Bauer H.C., Steiner M., Malfertheiner A., Lipp A.T. (2022). Lipedema research ‒ quo vadis?. J Pers Med..

[22] Amato A.C.M., Saucedo D.Z., Santos K. da S., Benitti D.A. (2021). Ultrasound criteria for lipedema diagnosis. Phlebology..

[23] Amato A.C.M., Peclat A.P.R.M., Kikuchi R., Souza A.C. de, Silva M.T.B., Oliveira R.H.P. de (2025). Brazilian consensus statement on lipedema using the Delphi methodology. J Vasc Bras..

[24] Bertsch T., Erbacher G., Elwell R., Partsch H. (2020). Lipoedema: a paradigm shift and consensus. J Wound Care..

[25] Torre Y., Wadeea R., Rosas V., Herbst K. (2018). Lipedema: friend and foe. Horm Mol Biol Clin Investig..

[26] Clarke C., Kirby J.N., Smidt T., Best T. (2023). Stages of lipoedema: experiences of physical and mental health and health care. Qual Life Res..

[27] Fu M.R., Ridner S.H., Hu S.H., Stewart B.R., Cormier J.N., Armer J.M. (2013). Psychosocial impact of lymphedema: a systematic review of literature from 2004 to 2011. Psychooncology..

[28] Dudek J.E., Białaszek W., Ostaszewski P. (2016). Quality of life in women with lipoedema: a contextual behavioral approach. Qual Life Res..

[29] Buso G., Favre L., Vionnet N., Gonzalez-Rodriguez E., Hans D., Puder J.J. (2022). Body composition assessment by dual-energy X-Ray absorptiometry: a useful tool for the diagnosis of lipedema. Obes Facts..

[30] Kumar P., Khan I.A., Das A., Shah H. (2022). Chronic venous disease. Part 1: pathophysiology and clinical features. Clin Exp Dermatol..

[31] Rossi A.B.R., Vergnanini A.L. (2000). Cellulite: a review. J Eur Acad Dermatol Venereol..

[32] Herbst K.L. (2012). Rare adipose disorders (RADs) masquerading as obesity. Acta Pharmacol Sin..

[33] Gabriel A., Chan V., Caldarella M., Wayne T., O’Rorke E. (2023). Cellulite: current understanding and treatment. Aesthet Surg J Open Forum..

[34] Arora G., Patil A., Hooshanginezhad Z., Fritz K., Salavastru C., Kassir M. (2022). Cellulite: presentation and management. J Cosmet Dermatol..

[35] Khalil S., Galadari H.I. (2024). Cellulite: an update on pathogenesis and management. Dermatol Clin..

[36] Hexsel D.M., Dal’Forno T., Hexsel C.L. (2009). A validated photonumeric cellulite severity scale. J Eur Acad Dermatol Venereol..

[37] Amato A.C.M. (2020). Is lipedema a unique entity?. Open J Clin Med Case..

[38] Jastreboff A.M., Aronne L.J., Ahmad N.N., Wharton S., Connery L., Alves B. (2022). SURMOUNT-1 Investigators. Tirzepatide once weekly for the treatment of obesity. N Engl J Med..

[39] Cifarelli V. (2025). Lipedema: progress, challenges, and the road ahead. Obes Rev..

[40] Peprah K.M.D. (2019). Liposuction for the treatment of lipedema: A review of clinical effectiveness and guidelines [Internet]. https://www.ncbi.nlm.nih.gov/books/NBK545818/.

[41] Bejar-Chapa M., Rossi N., King N., Hussey M.R., Winograd J.M., Guastaldi F.P.S. (2024). Liposuction as a treatment for lipedema: a scoping review. Plast Reconstr Surg Glob Open..

[42] Savoia A., Landi S., Vannini F., Baldi A. (2013). Low-level laser therapy and vibration therapy for the treatment of localized adiposity and fibrous cellulite. Dermatol Ther (Heidelb)..

[43] Amato A.C.M., Benitti D.A. (2021). Lipedema can be treated non-surgically: a report of 5 cases. Am J Case Rep..

[44] Ingargiola M.J., Motakef S., Chung M.T., Vasconez H.C., Sasaki G.H. (2015). Cryolipolysis for fat reduction and body contouring. Plast Reconstr Surg..

[45] Resende L., Noites A., Amorim M. (2022). Application of cryolipolysis in adipose tissue: a systematic review. J Cosmet Dermatol..

[46] Mulholland R.S., Paul M.D., Chalfoun C. (2011). Noninvasive body contouring with radiofrequency, ultrasound, cryolipolysis, and low-level laser therapy. Clin Plast Surg..

[47] Sadick N., Magro C. (2007). A study evaluating the safety and efficacy of the VelaSmooth system in the treatment of cellulite. J Cosmet Laser Ther..

[48] Fatemi A., Kane M.A. (2010). High-intensity focused ultrasound effectively reduces waist circumference by ablating adipose tissue from the abdomen and flanks: a retrospective case series. Aesthetic Plast Surg..

[49] Jewell M.L., Solish N.J., Desilets C.S. (2011). Noninvasive body sculpting technologies with an emphasis on high-Intensity focused ultrasound. Aesthetic Plast Surg..

[50] Choi S.Y., No Y.A., Kim S.Y., Kim B.J., Kim M.N. (2016). Tightening effects of high-intensity focused ultrasound on body skin and subdermal tissue: a pilot study. J Eur Acad Dermatol Venereol..

[51] Nomoto M., Narita M. (2024). What can ULTRAcel Q+ achieve? Treatment approach to skin. [Internet]. https://joa.jeisys-inc.com/blog/2024/02/28/505/.

[52] Knobloch K., Kraemer R. (2015). Extracorporeal shock wave therapy (ESWT) for the treatment of cellulite – A current metaanalysis. Int J Surg..

[53] Michelini S., Musa F., Vetrano M., Santoboni F., Nusca S.M., Latini E. (2023). Defocused and radial shock wave therapy, mesotherapy, and Kinesio taping effects in patients with lipedema: a pilot study. Lymphology..

[54] Amato A.C., Amato J.L., Benitti D. (2024). Efficacy of liposuction in the treatment of lipedema: a meta-analysis. Cureus..

